# Isolation and characterization of novel lytic bacteriophages against (fluoro)quinolone-resistant *Campylobacter* strains

**DOI:** 10.3389/fmicb.2025.1722119

**Published:** 2026-01-02

**Authors:** Yuran Elías Calancha-Padrón, Dayana Perez-Condori, Marcelo Fernando Gutierrez-Valverde, Daniel Martin Salas-Veizaga, Karin Hjort, María Teresa Alvarez-Aliaga

**Affiliations:** 1 Área de Bioquímica Molecular, Instituto de Investigaciones Fármaco Bioquímicas, Universidad Mayor de San Andrés, La Paz, Bolivia; 2 Department of Medical Biochemistry and Microbiology, Biomedicinskt Centrum, Uppsala University, Uppsala, Sweden

**Keywords:** bacteriophages, *Campylobacter* spp., antimicrobial resistance, (fluoro-)quinolone resistance, lytic activity

## Abstract

Antimicrobial resistance (AMR) has become a global public health concern, particularly in developing countries where antibiotics are often overused and misused. In Bolivia, the indiscriminate use of antibiotics, including (fluoro-)quinolones, has led to the proliferation of multidrug-resistant (MDR) *Campylobacter* spp., increasing the risk of resistance gene dissemination to other bacteria, and further deepening the AMR problem. To help mitigate the proliferation of MDR bacteria, bacteriophages can be a valuable complementary treatment to antibiotics. In the present study, we isolated and characterized three novel lytic bacteriophages with activity against (fluoro-)quinolone-resistant *Campylobacter* isolates and *C. jejuni* strains. The isolated bacteriophages, BMBo_CjP_006, BMBo_CjP_007, and BMBo_CjP_009, belong to the class *Caudoviricetes* and possess a linear double-stranded DNA genome. Their genome size ranges from 59 to 77 kb, with a GC-content between 42 to 46%. The 90, 144, and 146 predicted coding sequences (CDSs) of the different bacteriophages did not encode any antibiotic resistance, virulence, or lysogenic-associated genes, confirming their genetic safety and lytic nature. The isolated bacteriophages showed a narrow host range and lytic activity against nine (fluoro-)quinolone-resistant *Campylobacter* spp., including *C. jejuni,* with lytic activity varying at MOIs from 0.1 to 100, dependent on bacteriophage and host isolate. In addition, the bacteriophages were stable across a pH range of 4 to 10 and a temperature range of −20 °C to 70 °C. These characteristics make them promising for biotechnological applications due to their lytic activity, lack of resistance and virulence genes, and potential utility for product preservation.

## 1 Introduction

Foodborne infections are one of the most common causes of hospitalization worldwide (Moye et al., 2018; Silva et al., 2011). Among these, campylobacteriosis, a zoonotic disease caused by *Campylobacter* spp., is frequently reported in humans due to the consumption of raw or undercooked poultry (EFSA and ECDC, 2019; Mourkas et al., 2019). *Campylobacter* have shown an increasing number of antimicrobial resistant isolates, especially toward fluoroquinolones, quinolones, and macrolides, which are frequently used in the clinical treatment of campylobacteriosis (Luangtongkum et al., 2009; Tang et al., 2017). Chickens serve as a reservoir for *Campylobacter* spp., and numerous species of this genus naturally colonize the gastrointestinal tract of wild and domestic birds (Parzygnat et al., 2024). Improper handling of chicken products can result in diarrheal outbreaks and *Campylobacter*-related food poisoning (Biswas et al., 2007; Kreling et al., 2020; Portes et al., 2023).

In countries such as Chile, Peru, Argentina, and Brazil, multidrug-resistant (MDR) *Campylobacter* strains were isolated from samples of poultry meat, domestic animals, and environmental sources, showing that food-producing animals and the food chain are steps in the transmission of fluoroquinolone-resistant *Campylobacter* strains (Fernández and Pérez-Pérez, 2016; Guernier-Cambert et al., 2021). In Bolivia, there is a lack of monitoring of *Campylobacter* in the food production chain, and the only published study detected *Campylobacter jejuni* and *Campylobacter coli* in the majority of raw chicken meat samples tested (approximately 70%) was conducted by Condori Ticona et al. (2024). However, there are no studies on antimicrobial resistance profiles in *Campylobacter* in Bolivia. In addition, the data portal on antimicrobial use in animals intended for human consumption indicates that Bolivia lacks regulations and legislation governing the use of antimicrobials as growth promoters in agricultural production (Trinchera et al., 2025). To reduce *Campylobacter* contamination, various intervention strategies have been employed, including competitive exclusion and the use of chemical additives or antibiotics (Atterbury et al., 2003; Shin et al., 2001). These strategies are costly and often insufficient to prevent *Campylobacter* infection. In addition, the use of antibiotics leads to increased antibiotic resistance (Tang et al., 2023). Alternative treatments, such as the application of probiotics or the use of bacteriophages to reduce *Campylobacter* in the guts of chickens, show promising results (Abedon et al., 2021; Xue et al., 2023).

Bacteriophages are viruses that specifically infect bacteria, often with a narrow species host range, making them valuable biological agents for the treatment of infections caused by MDR bacteria, since their application will affect only the targeted bacteria and no other potential beneficial bacterial species (Murray et al., 2021). This characteristic makes them a suitable option since they do not indiscriminately kill a broad range of bacterial species (Abdelrahman et al., 2022; Yazdi et al., 2020). Bacteriophages are usually isolated directly from the same bacterial source and subsequently applied in medical, industrial, or environmental settings, yielding positive results (Chinivasagam et al., 2020; Kolenda et al., 2020; Schwarz et al., 2022). Several studies have shown that bacteriophages can be used mainly towards pathogens from the ESKAPE group and foodborne pathogens, including *Salmonella* spp., *Escherichia coli*, *Listeria monocytogenes*, *Clostridium perfringens,* and *C. jejuni* (Fischer et al., 2013; Steffan et al., 2021; Vaitekenas et al., 2021). An experimental study with *Campylobacter* phages has shown that they can effectively reduce *C. jejuni* loads *in vitro* across a range of pH and temperature conditions, demonstrating their robustness and potential for food safety applications (Steffan et al., 2021). Furthermore, *in vivo* studies indicate that phage cocktails reduce bacterial counts in broiler feces by 2–3 log 10 units and, in addition, prevent the emergence of antibiotic-resistant strains (Peh et al., 2023).

This study aimed to isolate and characterize novel lytic bacteriophages acting against (fluoro-)quinolone-resistant *Campylobacter* spp., including *C. jejuni,* from chicken feces samples.

## 2 Methods

### 2.1 Isolation and antimicrobial resistance determination of *Campylobacter* spp.

*Campylobacter* spp. were isolated from chicken feces collected at popular markets in El Alto, La Paz, Bolivia. Samples of chicken feces were resuspended in 0.9% (w/v) NaCl to obtain a 10-fold dilution of the sample. The diluted samples were filtered through 0.45-μm nitrocellulose membranes, and the membranes were subsequently placed on CHROMagar *Campylobacter* (CHROMagar, France) plates. The plates were incubated in a microaerophilic atmosphere at 42 °C for 48 h. Single red colonies were inoculated in 5 mL Brain Heart Infusion (BHI, OXOID, United Kingdom), followed by incubation at 42 °C for 48 h. For further purification of the bacterial isolates, 100 μL bacterial suspension was spread on CHROMagar *Campylobacter* plates, and the isolated colonies were re-streaked on blood agar plates for isolation. Finally, for preservation purposes at −80 °C, the resulting *Campylobacter* isolates were resuspended in BHI supplemented with 10 mM MgSO_4_, adding glycerol to a final concentration of 25% (v/v).

For the preparation of bacterial suspension, *Campylobacter* spp. were preserved at −80 °C, cultured on blood agar, and incubated under microaerophilic conditions at 42 °C for 48 h. The colonies formed were resuspended in NaCl (0.9% w/v) and adjusted to OD_600_ 0.3 (~1 × 10^8^ CFU/mL).

The Disk Diffusion Test (Oxoid, United Kingdom) was used to determine the phenotypic antimicrobial susceptibility of all 55 *Campylobacter* isolates, following the Clinical and Laboratory Standards Institute CLSI M45 methods for antimicrobial susceptibility testing of infrequently isolated or fastidious bacteria (CLSI, 2016). Antibiotic discs used included ciprofloxacin (CIP, 5 μg), nalidixic acid (NAL, 30 μg), ampicillin (AMP, 10 μg), amoxicillin/clavulanic acid (AMC, 20/10 μg), cefepime (CEP, 30 μg), tetracycline (TET, 30 μg), erythromycin (ERY, 15 μg), chloramphenicol (CHL, 30 μg), and sulfamethoxazole trimethoprim (SXT, 30 μg). MDR *Campylobacter* strains are defined as bacterial isolates resistant to at least three different classes of antibiotics (such as fluoroquinolones, macrolides, tetracyclines, and aminoglycosides). ResFinder was used to identify genotypic antimicrobial resistance genes within 14 sequenced *Campylobacter* strains (Bortolaia et al., 2020).

### 2.2 Enrichment and isolation of lytic bacteriophages

For the enrichment and isolation of bacteriophages, 2 g of a chicken feces sample was dissolved in 10 mL of SM buffer [50 mM Tris–HCl, pH 7.5, 100 mM NaCl, 8 mM MgSO_4_, 0.01% gelatin (w/v)] and incubated overnight at 42 °C with shaking (120 *rpm*). Enrichment of bacteriophages was done by mixing 5 mL of filtered chicken feces suspension (filtered through 0.22-μm nitrocellulose membranes) and 5 mL of 3 × 10^8^ CFU/mL suspension of *C. jejuni* strain Cj2. This strain was selected based on its abundant growth on blood agar plates. The filtrate was centrifuged at 13,000 *g* for 10 min at 4 °C and filtered through a 0.22-μm nitrocellulose membrane (Gencay et al., 2017). The filtrate was incubated under microaerophilic conditions at 42 °C and shaken at 120 *rpm* for 48 h. Bacteriophage isolation was performed according to Sørensen et al. (2017), and after 24 h of incubation, the resulting plaques were selected and resuspended in 1 mL of SM buffer. Purification of single plaques was repeated two more times.

### 2.3 Spot test

To determine the titer of isolated bacteriophages, a double-layer agar was used. *Campylobacter* isolates were streaked onto blood agar plates and incubated under microaerophilic conditions at 42 °C for 48 h. A few colonies from the agar plate were resuspended in 0.9% NaCl to reach an OD_600_ of 0.3 (~1 × 10^8^ CFU/mL). From the *Campylobacter* suspension, 200 μL was mixed with 6 mL of 0.6% NZCYM (Sigma-Aldrich, Germany) soft agar at 45 °C and poured over an agar plate (20 mL of 1.2% (w/v) NZCYM). On top of the *Campylobacter* layer, 10 μL of serial 10-fold dilutions of bacteriophages in the SM buffer were placed as described by Sørensen et al. (2017). The plates were incubated under microaerophilic conditions at 42 °C, and after 24 h, the plaques were counted to determine the plaque-forming units (PFU)/mL.

### 2.4 Morphology characterization of isolated bacteriophages using TEM

The morphology of the isolated bacteriophages was examined using transmission electron microscopy (TEM), following the method of Ackermann (2009) with some modifications. An enriched bacteriophage suspension of approximately 10^9^–10^10^ PFU/mL was fixed with an equal volume of 4% formaldehyde, and 5 μL of the sample was placed on a formvar- and carbon-coated 200-mesh copper grid (Ted Pella). The excess solution was removed by blotting with filter paper. The grid was then washed twice with drops of ultrapure water, followed by contrasting twice with a drop of 2% uranyl acetate. The excess of uranyl acetate was removed by blotting with filter paper, and the grid was air-dried. Images were acquired using a Tecnai G2 Spirit BioTwin TEM (Thermo Fisher/FEI, United States) at 80 kV, equipped with an ORIUS SC200 CCD camera and Gatan Digital Micrograph software (both from Gatan Inc./Blue Scientific). The identification and classification were performed according to the guidelines of the International Committee on Taxonomy of Viruses (ICTV) (Turner et al., 2023).

### 2.5 *Campylobacter* and bacteriophages DNA extraction

Genomic DNA from 14 *Campylobacter* isolates was extracted using the Wizard^®^ Genomic DNA Purification Kit (Promega, United States). For phage DNA extraction, the Phage DNA Isolation Kit (Norgen Biotek Corporation, Canada) was used, following the manufacturer’s instructions. The DNA quality was determined by measuring the OD_260_/OD_280_ and OD_260_/OD_230_ ratios spectrophotometrically (DeNovix, United States). DNA samples were stored at −80 °C until sequenced.

### 2.6 Whole-genome sequencing analysis of *Campylobacter* isolates and bacteriophages

Based on the phenotypic resistance profile, 13 out of the 55 *Campylobacter* isolates were selected, based on resistance toward nalidixic acid (NAL, quinolone), ciprofloxacin (CIP, fluoroquinolone), or both. In addition, one non-quinolone (fluoroquinolone) resistant *Campylobacter* isolate was also sequenced. The 14 *Campylobacter* isolates and the 3 lytic bacteriophages were sequenced using the MiSeq (Illumina, United States), performed in-house. For this, the libraries were prepared using the Nextera XT DNA library preparation kit, and sequencing was done with a V3 600-cycle reagent cartridge. The quality and trimming of raw reads were assessed using Fastp v0.24.0 (Chen, 2023), and reads were coverage-assembled using SPAdes genome assembler v.4.1.0 (metaviralSPAdes/viralVerify mode) (Antipov et al., 2020) using the default settings. The functional annotation, search for antibiotic resistance genes (*Campylobacter* and bacteriophages), and virulence factors (bacteriophages) were conducted using the VFDB and CARD databases with the bioinformatic tool Pharokka v1.7.0 (Bouras et al., 2023). Also, for the bacteriophages, genes related to lysogenic or lytic activity were determined by looking for integrases, recombinases, CI (Lambda repressor), CII (transcription activators), Cro (which encodes a repressor protein), PRM (CI transcription promoter), PR (lytic promoter), and PL (lytic promoter) in the bacteriophage genomes. For the identification of the closest bacteriophage genome publicly available, the Pharokka tool was used. To confirm the presence of virulence genes within bacteriophage genomes (lytic capability), the Phage. AI web service^1^ was used. Finally, the obtained sequences were BLASTed at the NCBI online service (United States). Based on ICTV taxonomic criteria classification (Siddell et al., 2023; Turner et al., 2023), to determine the identity of the bacteriophage, genetic identity and genomic coverage analyses of the phage genomes were performed using the Map to Reference tool in the Geneious Prime 2026.0 platform^2^.

### 2.7 Determination of bacteriophage host range

The host range of isolated bacteriophages was tested against all 55 *Campylobacter* isolates (isolated in this project), plus *Escherichia coli* ATCC 25922, *Staphylococcus aureus* ATCC 25923, *Klebsiella pneumoniae* BMBo_Kp_001, *Pseudomonas aeruginosa* BMBo_Pa_001, and *Enterococcus faecalis* BMBo_Ef_001 (obtained from the strain culture collection of the Instituto de Investigaciones Fármaco Bioquímicas, La Paz, Bolivia). Bacterial cultures (1 × 10^6^ CFU/mL) were seeded on Petri dishes containing 20 mL of NZCYM agar (1.2%) and further inoculated with 5 μL drops of propagated bacteriophages (1 × 10^8^ PFU/mL) placed on top, following the protocol established by Rahimzadeh et al. (2016). Then, the agar plates were incubated under microaerophilic conditions at 42 °C for 24 h; the next day, halos were observed on the bacterial lawn.

### 2.8 One-step growth curve

The one-step growth curve assay was performed according to the procedure of Pajunen et al. (2000) with minor modifications. Briefly, the *C. jejuni* strain Cj2 was cultured under the conditions described above and resuspended to an OD_600_ of 0.3. The bacterial suspension was then infected with the three bacteriophage samples separately at a multiplicity of infection (MOI) of 1, and the bacteriophages were allowed to adsorb for 10 min at room temperature. After adsorption, the mixture was centrifuged at 14500 *g* for 2 min, and the resulting pellet was resuspended in 10 mL of BHI medium. The mixture was then incubated at 42 °C for 150 min. At 10-min intervals, 200 μL aliquots were collected, serially diluted, and immediately plated for phage titration to determine the number of phage particles released from the infected bacterial cells. From the one-step growth curves, the latency period and burst size were determined.

### 2.9 Bacteriophage temperature and pH stability

For pH stability, 100 μL of phage suspension (10^5^ PFU/mL) was mixed with 900 μL of PBS (100 mM) adjusted with HCl [1 M] or NaOH [1 M] to achieve the following pH: 3, 4, 5, 7, 8, 10, 12. The samples were incubated at 37 °C for 60 min (Chen et al., 2023). As a control, bacteriophages in SM buffer (pH 7.5) were incubated for 60 min at 37 °C. For temperature stability, 100 μL of phage suspension (10^5^ PFU/mL) was mixed with 900 μL of PBS (100 mM) and incubated at −20, 50, 60, 70, and 80 °C in pH 7.5; as a control, bacteriophages were incubated at room temperature for 60 min (Chen et al., 2023). After 60 min of incubation, 100 μL from each condition was mixed with 900 μL of 100 mM SM buffer, pH 7.5. Subsequently, each phage sample from each condition was tested to determine the PFU/mL using the Spot Test, as described above. To fit all the pH and temperature assays in a single Petri dish per parameter, and to facilitate counting of halos, 10^5^ PFU/ml was selected as the initial phage concentration.

### 2.10 Bacteriolytic phage efficiency in terms of MOI and AUC

To evaluate the lytic activity of the isolated bacteriophages, the protocol of Steffan et al. (2021) was followed with some modifications. Isolates of *Campylobacter* spp. were streaked onto 5% (v/v) blood agar and incubated overnight at 42 °C. For the assay, one bacterial colony was resuspended in 1 mL MgSO_4_ [10 mM] and further diluted to 1.5 × 10^6^ CFU/mL in BHI. The bacterial suspension was mixed with the isolated bacteriophage at the following MOI: 100, 10, 1, and 0.1 in a 1:1 ratio. 500 μL of the 1:1 (bacteria:bacteriophage) mix was added to a 48-well microplate and incubated in microaerophilic conditions at 42 °C and 120 *rpm*. To determine the lytic effect, OD_600_ was measured every hour for 24 h (EPOCH Biotek, Agilent Technologies, United States). As a positive control, a 1:1 mix of bacterial suspension and SM buffer was used. As a negative control, a 1:1 mix of BHI and SM buffer was used. As a phage purity control, a 1:1 mix of BHI and Phage suspension was used. Triplicate measurements were performed in the experiment.

The area under the curve (AUC) represents bacterial growth over time, enabling the quantification of phage-induced bacterial lysis. The AUC was used as an index of lytic activity by comparing the AUC of the bacteriophage-treated samples at different MOIs against the positive growth control. The statistical significance was determined using the Student’s *t*-test with a 0.95 confidence interval. The visualization and statistical analysis were done using libraries: ggprism (v.1.0.4), DescTools (version 0.99.47), and Rstatix (v.0.7.2) in R software (v.1.4–25).

## 3 Results

A total of 55 *Campylobacter* spp. were isolated, and their phenotypic antimicrobial resistant profile was determined (Supplementary Table S1). Thirteen (fluro-)quinolone-resistant and one non-(fluro-)quinolone-resistant *Campylobacter* spp. were whole-genome sequenced and identified as *C. jejuni* based on genotypic 16S rRNA.

Genotypic antimicrobial resistance was also determined for the 14 sequenced *C. jejuni* isolates, and all of them had a *gyrA* (T86I) mutation, indicating ciprofloxacin and nalidixic acid resistance (Table 1). Furthermore, a mobile genetic element containing a *bla*_OXA-61_ gene was the most frequently detected resistance gene (13/14); one isolate contained the *bla*_OXA-452_ gene for betalactam antibiotics. The tetracycline-resistant strains contained the *tet*(O) gene (9/14) (Supplementary Table S1), and in addition, all sequenced strains harbored the multidrug efflux pump *cmeABC* (Lin et al., 2002).

**Table 1:** Host range, phenotypic and genotypic antimicrobial resistance profile of *C. jejuni* (Cj) and *Campylobacter* spp. (C) isolated from chicken feces at popular markets in La Paz and El Alto, Bolivia.

Bacterial isolate	Host range of the isolated bacteriophage			Phenotypic antimicrobial resistance	Genotypic antimicrobial resistance	Antimicrobial resistance gene(s)
	BMBo_CjP_006	BMBo_CjP_007	BMBo_CjP_009			
Cj1	+	−	+	AMP, CHL, NAL	CIP, NAL, AMP	*cmeABC, bla*_OXA-61_, *gyrA* (T86I)
Cj2	+	+	+	AMP, AMC, CHL, NAL, SXT	CIP, NAL, AMP	*cmeABC, bla*_OXA-61_, *gyrA* (T86I)
Cj3	+	−	−	AMP, TET, CHL, CIP, NAL	CIP, NAL, AMP	*cmeABC, bla*_OXA-61_, *gyrA* (T86I)
C4	+	−	−	AMP, CEP, TET, CHL, NAL	Not determined	
C5	−	+	−	CHL, NAL	Not determined	
Cj6	−	+	−	AMP, CHL, CIP	CIP, NAL, AMP, TET	*cmeABC, bla*_OXA-61_, *gyrA* (T86I), *tet(O)*
Cj9	−	+	−	AMP, CIP	CIP, NAL, AMP	*cmeABC, bla*_OXA-61_, *gyrA* (T86I)
C28	−	+	−	AMP, ERY, CHL, CIP, NAL	Not determined	
C45	−	+	−	AMP, AMC, CEP, CIP, NAL, SXT	Not determined	

AMP: Ampicillin; CHL: Chloramphenicol; NAL: Nalidixic Acid; AMC: Amoxicillin/Clavulanic Acid; SXT: Trimethoprim/Sulfamethoxazole; TET: Tetracycline; CEP: Cefepime; CIP: Ciprofloxacin; ERY: Erythromycin.

### 3.1 Three novel bacteriophages with lytic activity against (fluoro)quinolone *Campylobacter* spp. were isolated

**Figure 1:**
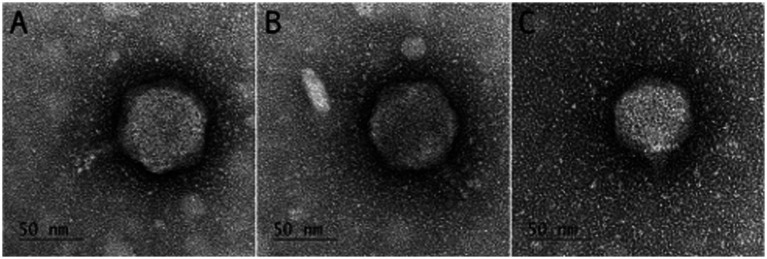
TEM images of bacteriophages **(A)** BMBo_CjP_006, **(B)** BMBo_CjP_007, and **(C)** BMBo_CjP_009 isolated from *C. jejuni*. Scale bars: 50 nm.

Three bacteriophages were isolated from chicken feces samples: BMBo_CjP_006, BMBo_CjP_007, and BMBo_CjP_009. These bacteriophages were characterized by short, non-contractile tails and icosahedral capsids (podoviruses), and a double-stranded DNA genome determined by TEM images and whole genome sequencing, respectively. According to ICTV parameters, they belong to the bacteriophage class of *Caudoviricetes* (Turner et al., 2023). These phages infect *Campylobacter* spp. and form characteristic plaques (small and clear) on the lawn of *Campylobacter* isolates, showing halo diameters ranging between 0.7 and 1.1 mm, measured using the Fiji Image Tool. The phage BMBo_CjP_006 possesses a tail with an average length of 33.3 nm and a capsid with an average length of 77.7 nm. For BMBo_CjP_007, the average length of the tail was 31.9 nm, and for the capsid, 73.9 nm. The phage BMBo_CjP_009 had an average tail length of 25.3 nm and 65.3 nm for the capsid (Figures 1A–C).

Regarding the host range, nine *Campylobacter* isolates were susceptible to lysis by the three bacteriophages (Table 1). The bacteriophages BMBo_CjP_006, BMBo_CjP_007, and BMBo_CjP_009 exhibited lytic activity against (fluoro-)quinolone-resistant *C. jejuni* and *Campylobacter* spp., which were either resistant to ciprofloxacin, nalidixic acid, or both (Table 1). None of the bacteriophages lysed *E. coli* ATCC 25922, *S. aureus* ATCC 25923, *K. pneumoniae* BMBo_Kp_001, *P. aeruginosa* BMBo_Pa_001, or *E. faecalis* BMBo_Ef_001.

### 3.2 Bacteriophage genomic analysis

The sequence analysis of the whole-genome sequences of the three bacteriophages revealed a linear, double-stranded DNA genome. The phage BMBo_CjP_006 (Accession Code: PX400320) genome size was 77,929 bp with a GC-content of 42%, and 146 CDSs were identified. The phage BMBo_CjP_007 (Accession Code: PX400315) was 76,852 bp in length, has a GC-content of 42%, and encodes 144 CDSs. Finally, the phage BMBo_CjP_009 (Accession Code: PX400321) has a length of 58,576 bp, 46.4% GC content, and 90 identified CDSs.

**Figure 2:**
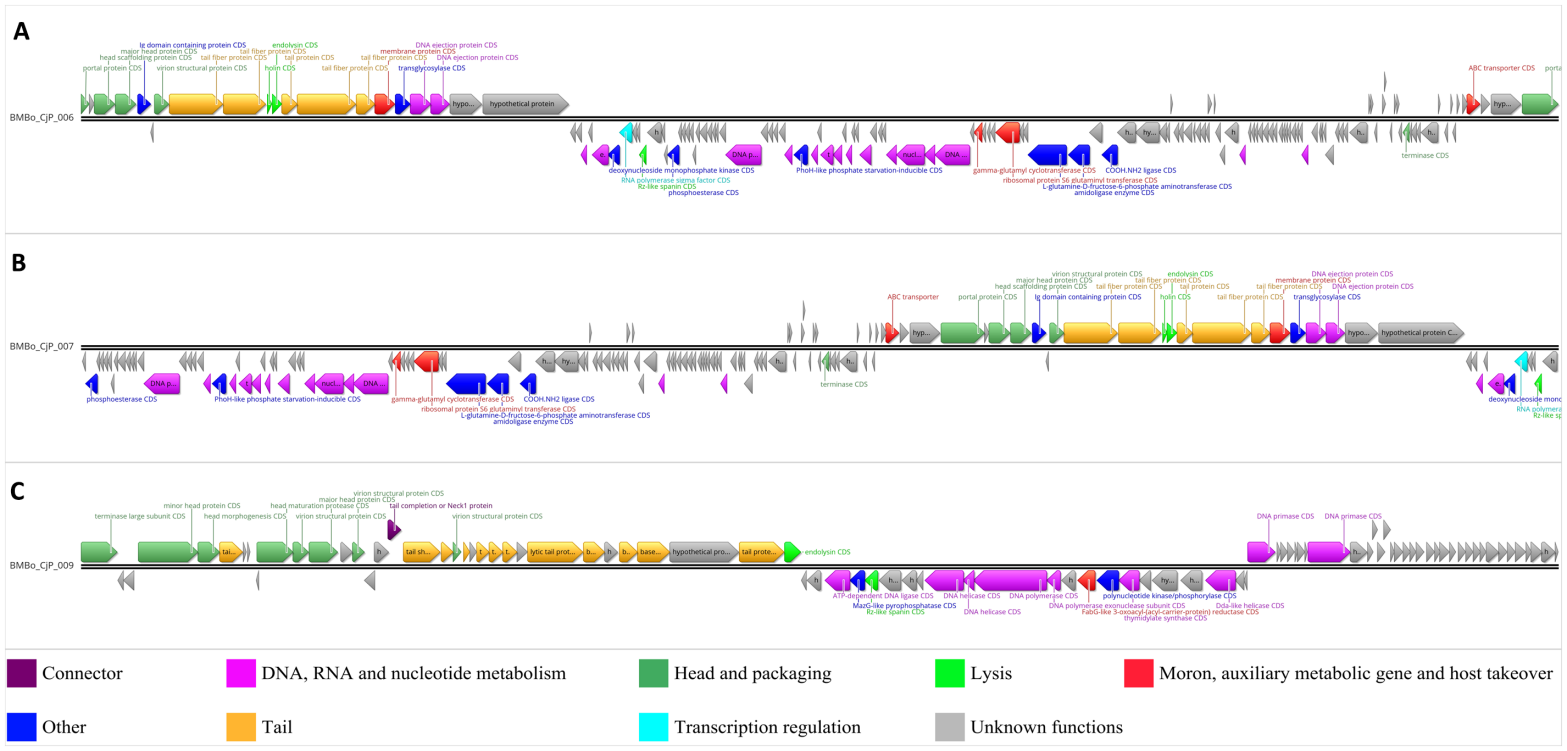
Graphic representation of functional annotations for the three bacteriophage genomes. The CDSs are color-coded according to nine functional groups of CDSs. Above each annotated CDS, an annotation is displayed according to the color coding of the functional group of CDS based on Pharokka. **(A)** Bacteriophage BMBo_CjP_006, **(B)** Bacteriophage BMBo_CjP_007, **(C)** Bacteriophage BMBo_CjP_009. A graphic representation is generated in Geneius Prime 2026.0 based on the Pharokka annotation report.

The functional annotation revealed that BMBo_CjP_006 (Figure 2) and BMBo_CjP_007 have approximately 30% CDSs with known function, with a similar number of coding sequences between them. For the bacteriophage BMBo_CjP_009, 39% of the CDSs have known functions. A detailed examination of the coding sequences identified several functional groups of genes, including (1) DNA, RNA, and nucleotide metabolism, (2) bacteriophage head and packaging, (3) lysis, (4) moron, auxiliary metabolic genes and genes involved in host takeover, (5) bacteriophage tail genes, (6) transcription regulation, (7) connector genes, (8) other genes, and (9) genes with unknown functions (Figure 2; Supplementary Table S2). The genes in the lytic gene category of the bacteriophage genome sequences are endolysins, spanins, and holins. No integration or excision genes were found in the three bacteriophage genomes, which fits well with their lytic nature. Furthermore, the virulence (lytic capability) of the bacteriophages was confirmed through the Phage. AI web service. In addition, no genes for antibiotic-resistant or virulence factors were found in the genomes, according to the virulence factor database (VFDB) (Chen et al., 2005) and the comprehensive antibiotic resistance database (CARD) (Alcock et al., 2023).

The genetic identity and genomic coverage analysis determined by BLASTn showed <95% nucleotide identities and coverage of <0.5% for BMBo_CjP_006 and BMBo_CjP_007, with respect to *Escherichia* phage AnnaHegner Bas87 (PQ850594). On the other hand, the results from Pharokka showed nucleotide identities of 88.7 and 84.0%, and coverages of 92.7 and 53.2% for BMBo_CjP_006 and BMBo_CjP_007, respectively, in comparison to Phage NC-B (MK310183). For BMBo_CjP_009, the nucleotide identity (95.3%) to the Shigella phage CWP003 (OL856099) and coverage (85.5%) were the same with both tools. Based on the ICTV criteria, none of the bacteriophages are identical to the closest identified phage sequences at the species taxonomic level.

### 3.3 The bacteriophages showed an even stability in a wide temperature and pH range

The stability range of lytic activity for different pH levels was from 4 to 10 for all bacteriophages, and the recovery of the bacteriophages was approximately 5-log_10_ PFU/mL, but at pH 12, no lytic activity was observed after 60 min of incubation. The three bacteriophages had an even stability of lytic activity at temperatures between −20 and 70 °C after 60 min, with a bacteriophage recovery similar to the control, approximately 3-log_10_ PFU/mL. None of the bacteriophages showed lytic activity after incubation at 80 °C (Supplementary Figure S1).

### 3.4 Determination of latent period and burst size

**Figure 3:**
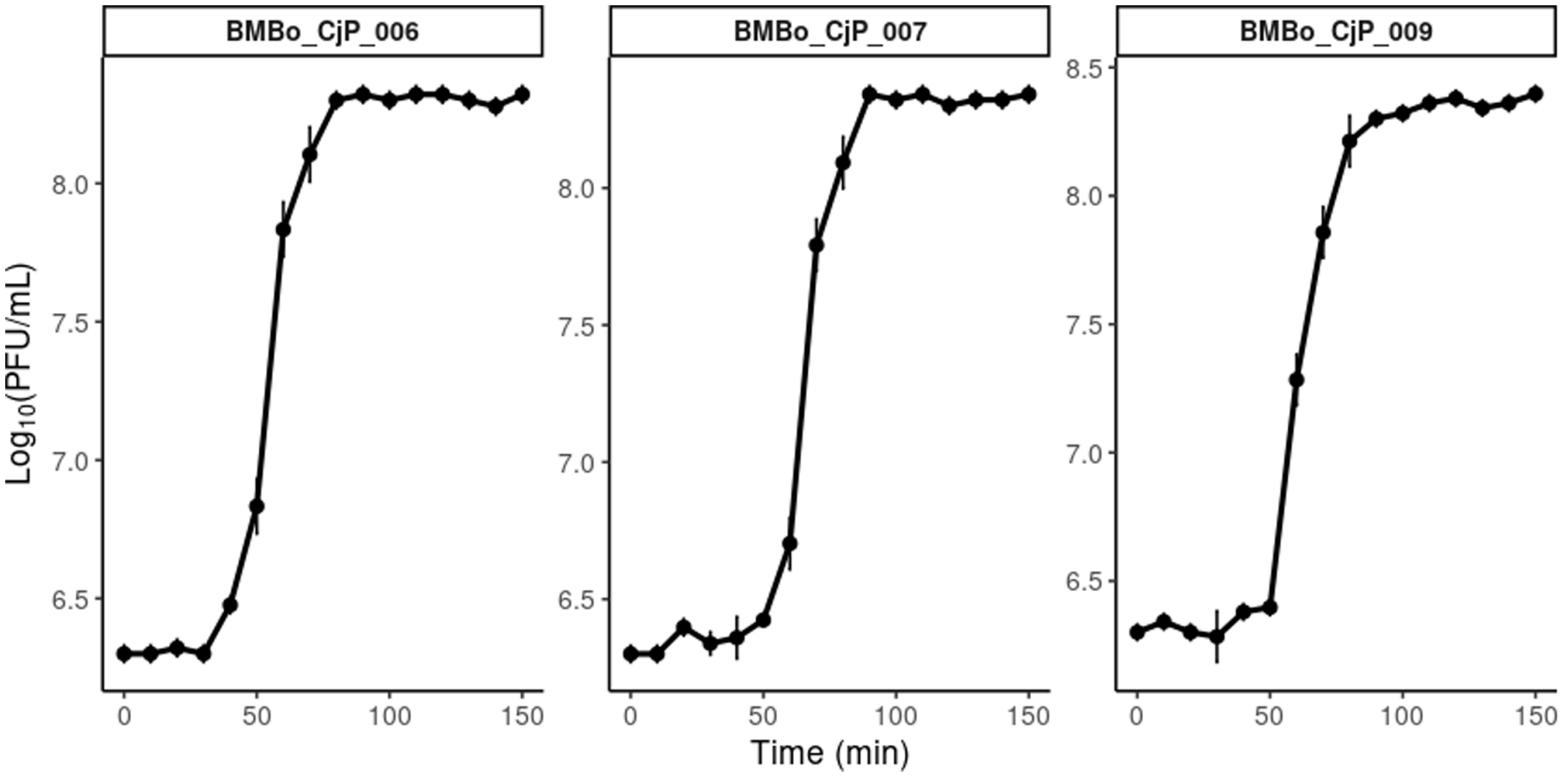
One-step growth curve of the three bacteriophages: BMBo_CjP_006, BMBo_CjP_007, and BMBo_CjP_009.

The one-step growth curve experiment was conducted to characterize the replication dynamics of *C. jejuni* phages BMBo_CjP_006, BMBo_CjP_007, and BMBo_CjP_009. The results showed that BMBo_CjP_006 had a latent period of 40 min, whereas BMBo_CjP_007 and BMBo_CjP_009 both had latent periods of 50 min. The burst sizes for BMBo_CjP_006, BMBo_CjP_007, and BMBo_CjP_009 were estimated to 92 ± 0.1, 94 ± 8, and 104 ± 2 new viral particles per infected cell, respectively (Figure 3).

### 3.5 Efficiency of the isolated lytic bacteriophages against (fluoro-)quinolone-resistant *Campylobacter* strains

**Figure 4:**
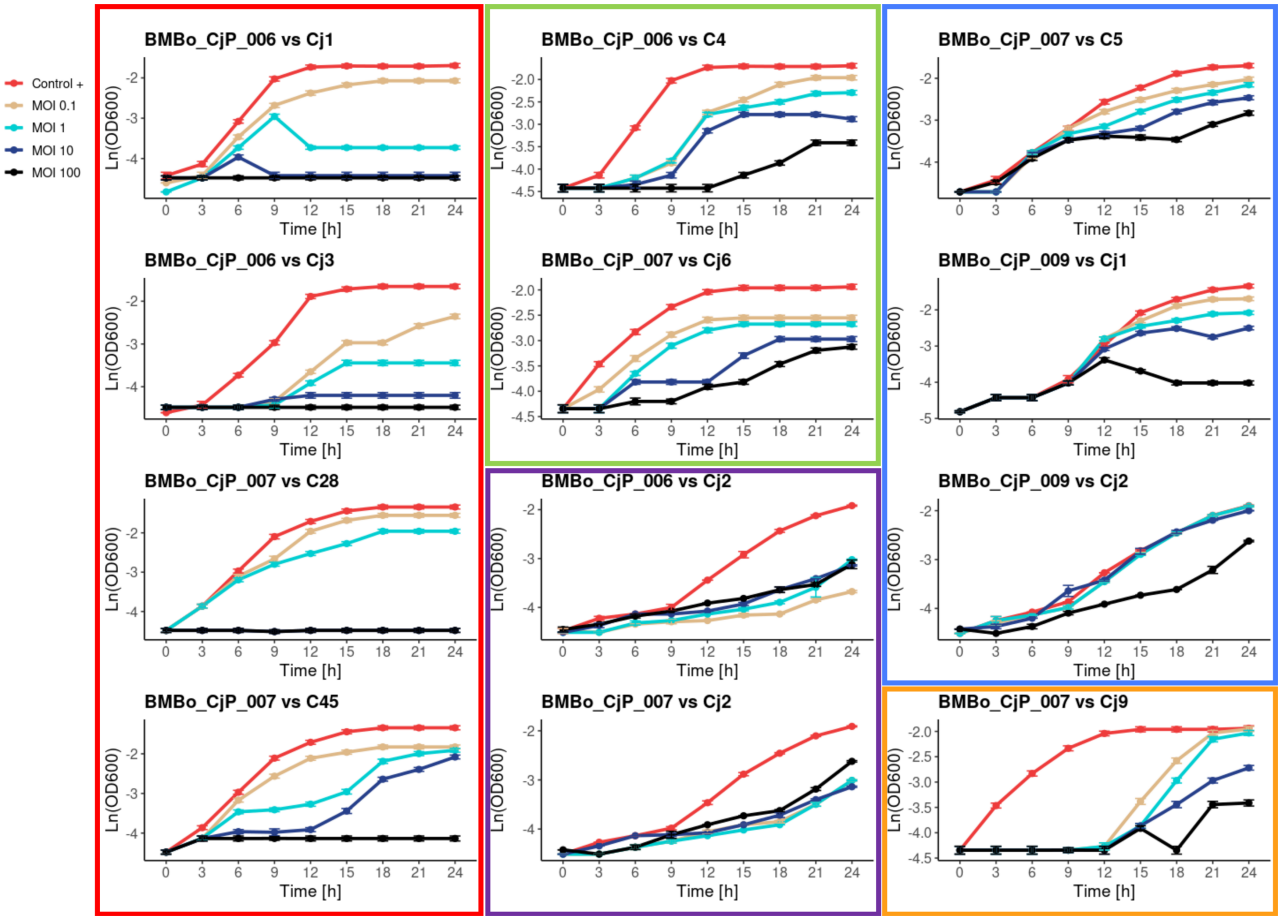
Growth kinetics for isolates of *C. jejuni* (Cj) and *Campylobacter* spp. (C): Cj1, Cj2, Cj3, Cj4, C5, Cj6, Cj9, C28, and C45 infected with bacteriophages BMBo_CjP_006, BMBo_CjP_007, and BMBo_CjP_009 at MOI 100, 10, 1, and 0.1. Triplicate samples are shown (average ± SD) in terms of Ln (OD_600_). The kinetics are separated into five different groups based on the growth inhibition curves of the bacterial hosts. Group 1 (red square) contains the *Campylobacter* isolates that are fully inhibited for 24 h at MOI 100 and 10. Group 2 (green square) contains grouped *Campylobacter* isolates with strains inhibition directly proportional to increasing the tested MOI. Group 3 (purple square) exhibits statistically significant growth inhibition of the *Campylobacter* isolates, compared to the control, but without total inhibition in the range of tested MOI. Group 4 (blue square) is *Campylobacter* isolates with a slight inhibition at MOI 100. Group 5 (orange square) shows a total growth inhibition of the *Campylobacter* isolate at all measured MOI for the first 12 h, with re-growth for the *Campylobacter* strains at all MOI analyzed.

**Figure 5:**
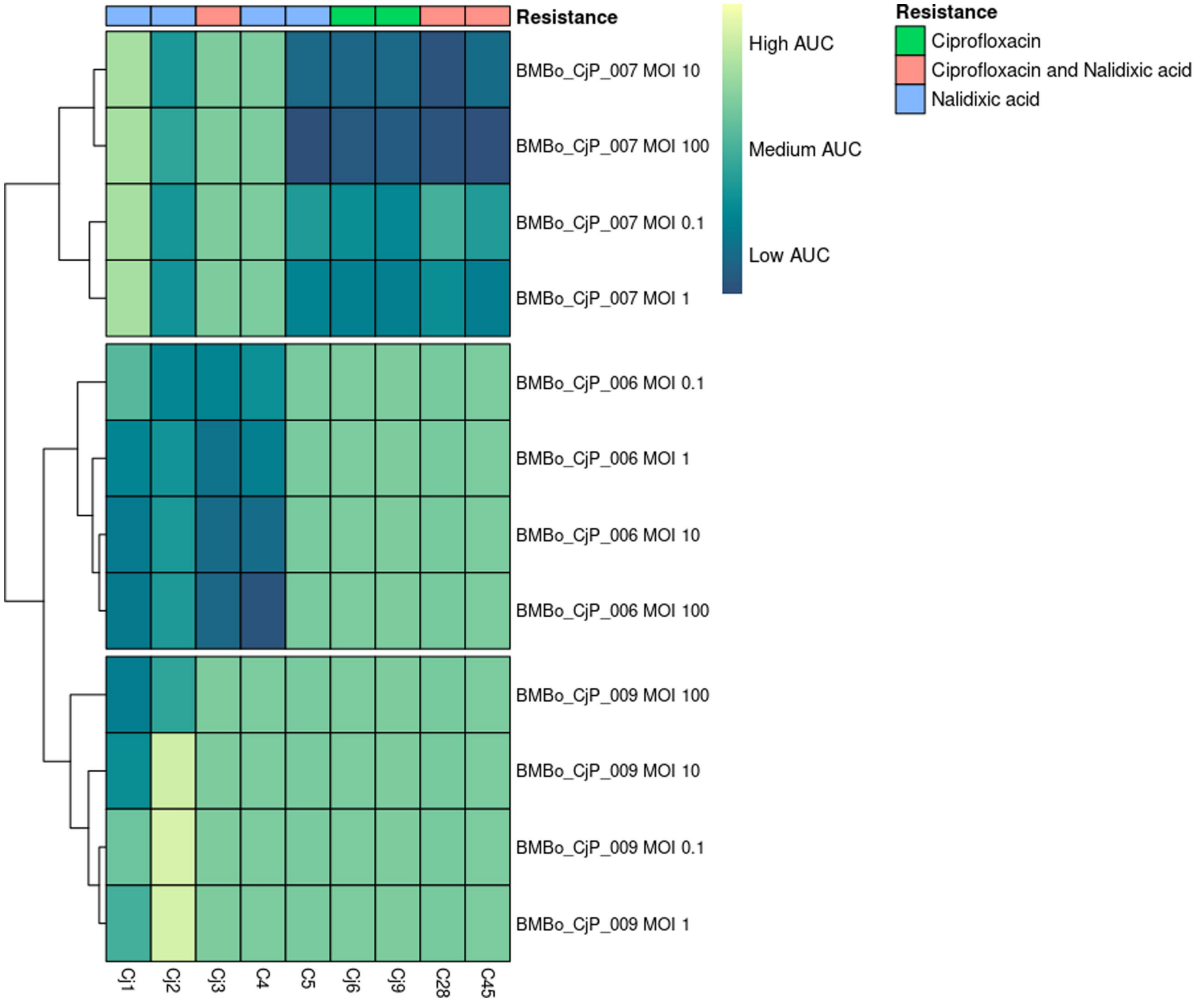
Heatmap of the area under the curve (AUC) values obtained from (fluoro-)quinolone-resistant isolates of *C. jejuni* and *Campylobacter* spp. treated with the respective bacteriophage (BMBo_CjP_006, BMBo_CjP_007, or BMBo_CjP_009) at different MOI (100, 10, 1, and 0.1). Antibiotic resistance phenotypic pattern of the *C. jejuni* and *Campylobacter* spp. is shown above the image. Bacteriophages are ordered from most effective to least effective against *Campylobacter* isolates. AUC is calculated based on the triplicate samples in the bacteriophage kinetic growth curves. High AUC is shown in yellow, medium AUC in green, and low AUC in dark green.

The lytic activity of the isolated bacteriophages was determined in terms of MOI and AUC. The MOI needed for lysis varied among the different bacteriophage:bacterial combinations. The results showed five different characteristics of lytic activity for the bacteriophages with respect to their *Campylobacter* host strain. The first group include BMBo_CjP_006 and BMBo_CjP_007 lysing NAL-resistant Cj1, CIP/NAL-resistant Cj3, and CIP/NAL-resistant C28 and C45 *Campylobacter* isolates, respectively. These bacteriophages exhibited a total inhibition of these *Campylobacter* isolates at MOIs of 10 and 100 (*p* < 0.05) (Figures 4, 5; Supplementary Figures S2, S3). However, at lower MOIs of 1 and 0.1, they showed a decrease in the bacterial growth rate for 24 h, but at the same time, the bacterial OD seemed to be equal to the control (*p* > 0.05). The second group encompasses BMBo_CjP_006 and BMBo_CjP_007 together with *Campylobacter* isolates NAL-resistant C4 and CIP-resistant Cj6 (*p* < 0.05), respectively. This group showed a clear difference in the inhibition directly proportional to the increasing MOI; however, the inhibition, even at an MOI of 100, was partial. The third group showed a statistically significant inhibition of the growth for NAL-resistant Cj2 in comparison to the growth control, using BMBo_CjP_006 and BMBo_CjP_007 phages (*p* < 0.05); however, there is no significant difference in the inhibition among all the MOI measured (Figures 4, 5). The fourth group showed a partial inhibition in the growth of the host at MOI 100 for BMBo_CjP_009 against NAL-resistant Cj1 and Cj2 strains, and for BMBo_CjP_007 against NAL-resistant C5 (*p* < 0.05), while the rest of the measured MOI showed partial or no inhibitory effect compared to the growth control. Finally, the fifth group showed BMBo_CjP_007 acting over CIP-resistant *C. jejuni* strain Cj9, and this bacteriophage showed a total inhibition of the growth of the host during the first 12 h of growth at all the measured MOI (*p* < 0.05). Interestingly, after this time, *C. jejuni* strain Cj9 started to grow at all MOI (Figures 4, 5).

## 4 Discussion

The phenotypic resistance profiles of all 55 *Campylobacter* spp. isolates were determined, and 82% (12 *C. jejuni* and 33 *Campylobacter* spp.) were classified as MDR, exhibiting phenotypic resistance to at least three different classes of antibiotics, including tetracyclines, aminoglycosides, fluoroquinolones, and macrolides (Supplementary Table S1). Moreover, 49 out of the 55 (89%) *Campylobacter* spp. isolates showed resistance to at least one fluoroquinolone or quinolone antibiotic (Supplementary Table S1). Although fluoroquinolone-resistant *C. jejuni* strains were previously categorized as high-priority pathogens by the World Health Organization (WHO, 2017), they are no longer included in the most recent list of critical priority pathogens (WHO, 2024). However, the need for surveillance of *C. jejuni* remains important in the South American region since this pathogen continues to be highly relevant in the region due to its persistent resistance to multiple antibiotics and its capacity to disseminate (fluoro-)quinolone resistance genes, as well as other antimicrobial resistance determinants, within microbial populations (Han et al., 2012; Portes et al., 2023). For instance, studies performed in Brazil (Buiatte et al., 2025), Argentina, Uruguay, Paraguay, Colombia, Trinidad & Tobago, Peru, Ecuador (Portes et al., 2023) and Chile (Bravo et al., 2021; Collado, 2020) showed similar levels of (fluoro-)quinolone resistance in *Campylobacter* as the data described here, highlighting the necessity of continuous monitoring of (fluoro-)quinolone resistant *Campylobacter* strains.

The mutation T86I in the *gyrA* gene present in all sequenced *Campylobacter* spp. is characteristic of high-level (fluoro-)quinolone resistance in *Campylobacter* isolates (El-Adawy et al., 2023). In addition, the presence of genes such as *bla*_OXA-61_ present on a mobile genetic element represents a potential risk of dissemination of antibiotic resistance to different environments, e.g., chicken feces (Griggs et al., 2009; Yanestria et al., 2023).

In this study, three bacteriophages, BMBo_CjP_006, BMBo_CjP_007, and BMBo_CjP_009, were isolated from chicken feces. The three bacteriophages were propagated in *C. jejuni* strain Cj2 and subsequently used to test host range among a set of *Campylobacter* spp., isolated from the same source. In fact, only *C. jejuni* (Cj2) was susceptible to all three bacteriophages, and *C. jejuni* (Cj1) was susceptible to two bacteriophages, BMBo_CjP_007 and BMBo_CjP_009. For the rest of the *Campylobacter* spp. and *C. jejuni,* only a few of them were sensitive to one of the bacteriophages, showing a narrow host range (Table 1). A narrow species host range of *Campylobacter* phages was also observed previously (Al-Mohammadi et al., 2022). The narrow species host range observed presents both advantages and disadvantages for a potential bacteriophage application (Golz and Stingl, 2021; Oechslin, 2018). On one hand, the advantage of a narrow species host range minimizes the risk of disrupting beneficial microbiota (Park et al., 2017), ensuring that only the target pathogen, *Campylobacter* spp., is affected. This specificity also reduces collateral damage compared to broad-spectrum antibiotics, which aligns well with *One Health* strategies, focusing on preserving microbial diversity. Moreover, it decreases the risk of unintended ecological consequences that might arise from non-specific lytic activity (Vaitekenas et al., 2021). On the other hand, a narrow host range toward only a few *Campylobacter* isolates can be considered a disadvantage due to the high diversity of *Campylobacter* species (Steffan et al., 2021; Ushanov et al., 2020).

The genome analysis of BMBo_CjP_006, BMBo_CjP_007, and BMBo_CjP_009 did not present any integration or excision genes (CI, CII, Cro, PRM, PR, and PL), confirming their lytic nature. In addition, the efficient replication cycles observed in the one-step growth curve (Figure 3), the absence of resistance or virulence genes in their genomes, and the preservation of their lytic activity after 60 min in a wide pH and temperature range (pH 4–10 and temperature −20 to 70 °C) make these bacteriophages potential candidates for the development of bioproducts. Only a few studies have shown stability in phage activity after incubation at 70 °C for 60 min. In a study by Rafiq et al. (2025), a 2-log reduction in PFU/mL was observed at 60 °C for 60 min without any detectable phages at 70 °C. To some degree, Al-Mohammadi et al. (2022) reported temperature tolerance of a *Campylobacter* phage at 70 °C, although this was accompanied by a 5-log reduction in PFU/mL. However, a study on *E. coli* bacteriophages reported a phage activity at 70 °C for 60 min without affecting the phage viability (Litt et al., 2018). Thermal stability is a crucial factor for bacteriophages used in food safety, particularly for processing steps, such as pasteurization or application of products at elevated temperatures.

The lytic efficiency of BMBo_CjP_006, BMBo_CjP_007, and BMBo_CjP_009 showed differences in the way they interact with their *Campylobacter* host strain. In that sense, five different patterns of *Campylobacter* inhibition were observed. The first pattern observed for bacteriophage BMBo_CjP_006 and BMBo_CjP_007 was a total inhibition of bacterial growth at MOI 100 toward CIP/NAL-resistant *C. jejuni* strains Cj1 and Cj3, and *Campylobacter* spp. isolates C28 and C45, respectively. This result suggests that these bacteriophages had a high rate of infection, inhibiting the bacterial growth for at least 24 h, decreasing the risk of *Campylobacter* to develop a mechanism of tolerance or resistance toward the bacteriophages (Abedon, 2023; Labrie et al., 2010). The second pattern was observed for BMBo_CjP_006 toward NAL-resistant *Campylobacter* spp. C4 and for BMBo_CjP_007 toward CIP-resistant *C. jejuni* Cj6, where the *Campylobacter* isolates showed a deceleration of the growth rate while the assayed MOI was increased; however, they did not show total inhibition of bacterial growth, even at MOI 100 (Supplementary Figure S3). The third pattern represents the lytic activity of BMBo_CjP_006 and BMBo_CjP_007 toward NAL-resistant *C. jejuni* Cj2, showing a statistically significant inhibition of the bacterial growth for all MOI assayed in comparison to the host growth control, but the lytic activity was the same among all measured MOI. Both patterns 2 and 3 may respond to a modification in the phage receptor sites, changing the injection site conformation and abolishing phage DNA entry to the cell (Fernández and Pérez-Pérez, 2016; Guernier-Cambert et al., 2021). The fourth pattern includes the lytic activity of BMBo_CjP_007 over NAL-resistant C5 and also BMBo_CjP_009 over NAL-resistant *C. jejuni* Cj1 and Cj2. In these growth kinetics, the same behavior, as described for the second pattern, is noticed, except that a partial cell growth inhibition was only shown at MOI 100, supporting the fact that the more bacteriophages, the more probability of phage infection (Labrie et al., 2010). The fifth pattern was observed for BMBo_CjP_007 toward CIP-resistant *C. jejuni* Cj9, where an effective inhibition of cell growth was clearly noticed during the first 12 h. However, after this time, a bacterial re-growth was observed, maybe due to an intracellular degradation of phage DNA by restriction modification systems described by *Campylobacter* strains (Ushanov et al., 2020) or phage resistance development (Labrie et al., 2010).

In conclusion, the three isolated bacteriophages showed a statistically significant inhibition effect against (fluoro-) quinolone-resistant *C. jejuni* and *Campylobacter* spp. Their genomes did not contain antimicrobial resistance, virulence, or lysogenic-associated genes, and the bacteriophages were host-specific toward *Campylobacter* spp. Furthermore, these bacteriophages also exhibited broad stability across different temperatures and pH levels, making them promising agents for biotechnological applications against MDR *Campylobacter* isolates, including *C. jejuni*.

## Data Availability

The datasets [bacteriophage genome sequences; NCBI accession numbers PX400320 (BMBo_CjP_006), PX400315 (BMBo_CjP_007), and PX400321 (BMBo_CjP_009) and *Campylobacter* isolates (NCBI project number PRJNA1336325)] for this study can be found in the (National Center for Biotechnology Information) (https://www.ncbi.nlm.nih.gov/).

## Footnotes

1
https://www.phage.ai/

2
http://www.geneious.com/
