# Supplementary material for: Isolation and characterization of novel lytic bacteriophages against (fluoro)quinolone-resistant Campylobacter strains
Source: Front Microbiol. 2026 Jan 2;16:1722119. doi: 10.3389/fmicb.2025.1722119 (PMC12808351; doi:10.3389/fmicb.2025.1722119)
Supplement: Supplementary file 1 [file Data_Sheet_1.docx]

***Supplementary Material***

# Supplementary Figures and Tables

## Supplementary Figures


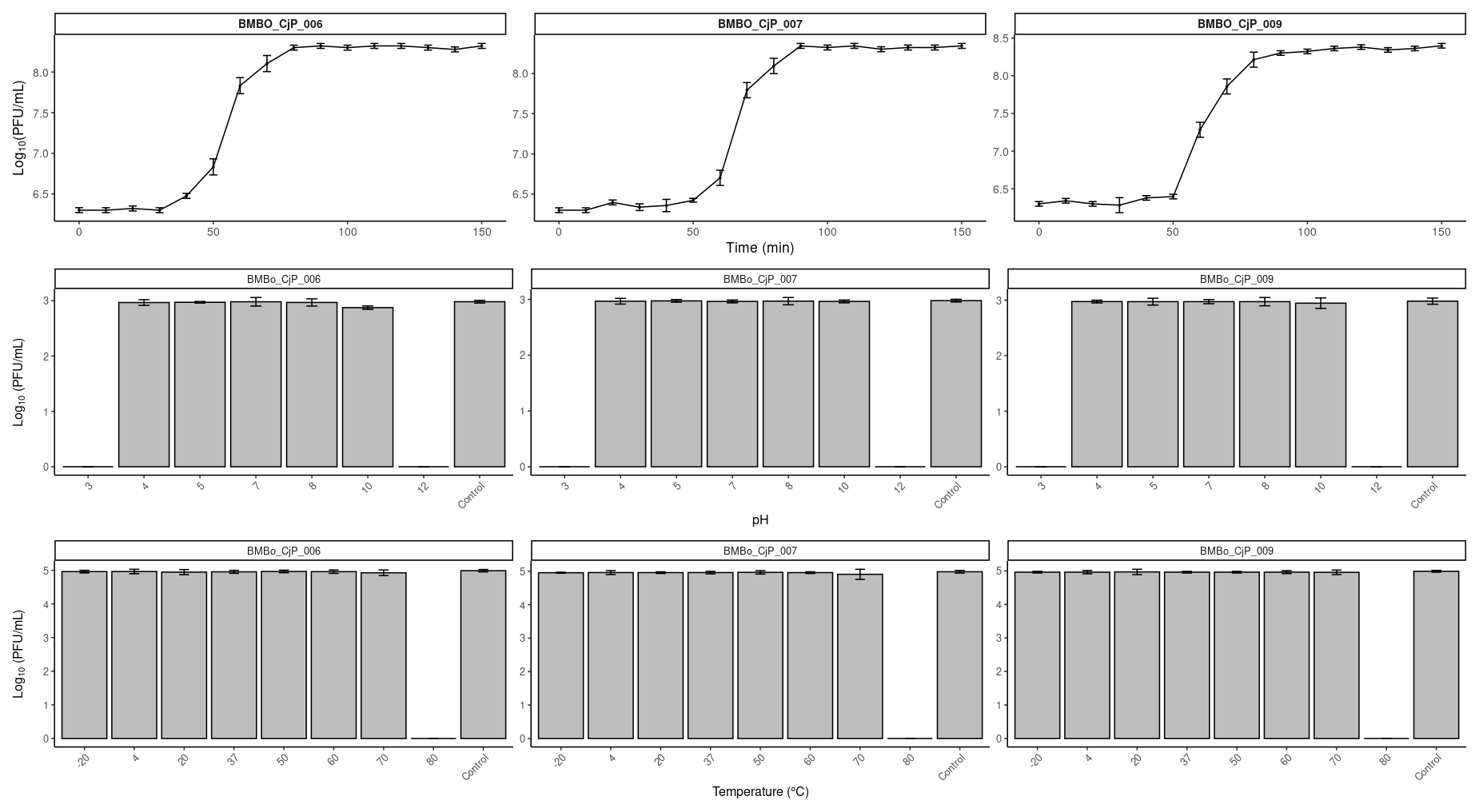


**Supplementary Figure 1.** Stability of bacteriophages BMBo_CjP_006, BMBo_CjP_007, and BMBo_CjP_009 determined as Log_10_ PFU/mL at different pH and temperature conditions in comparison with controls (pH control at pH 7.5 and temperature control at room temperature) after 1 h of incubation.


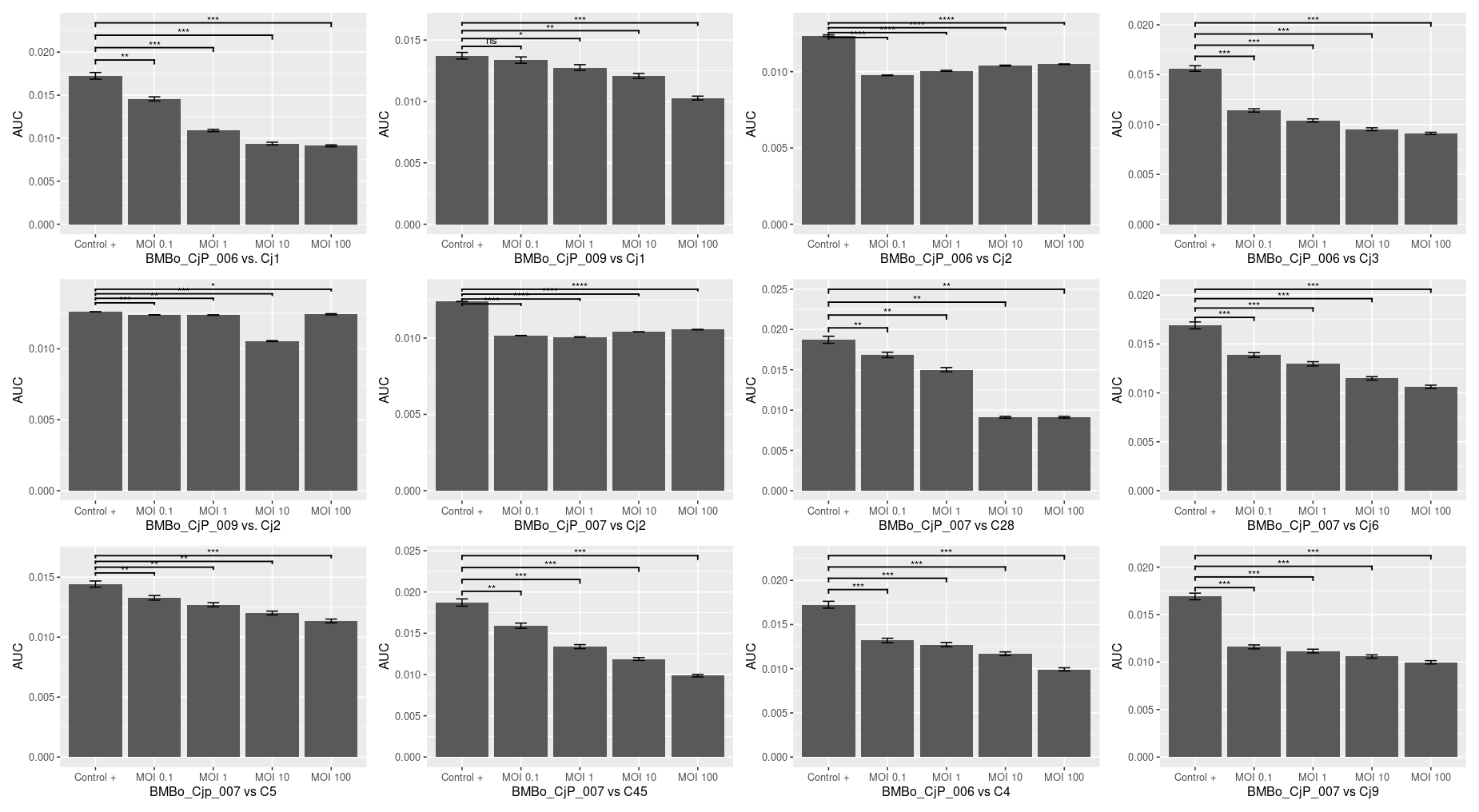
**Supplementary Figure 2.** Lytic activity of bacteriophages determined as area under the curve (AUC) for bacteriophages BMBo_CjP_006, BMBo_CjP_007, and BMBo_CjP_009 at different MOI (100, 10, 1, and 0.1) against Campylobacter *spp*. (Cj1, Cj2, Cj3, C4, C5, Cj6, Cj9, C28, C45). Student t-test was used to determine significant difference in comparison to the controls without bacteriophage added (p<0.01 = **; p<0.05 = *, ns = non-significant).


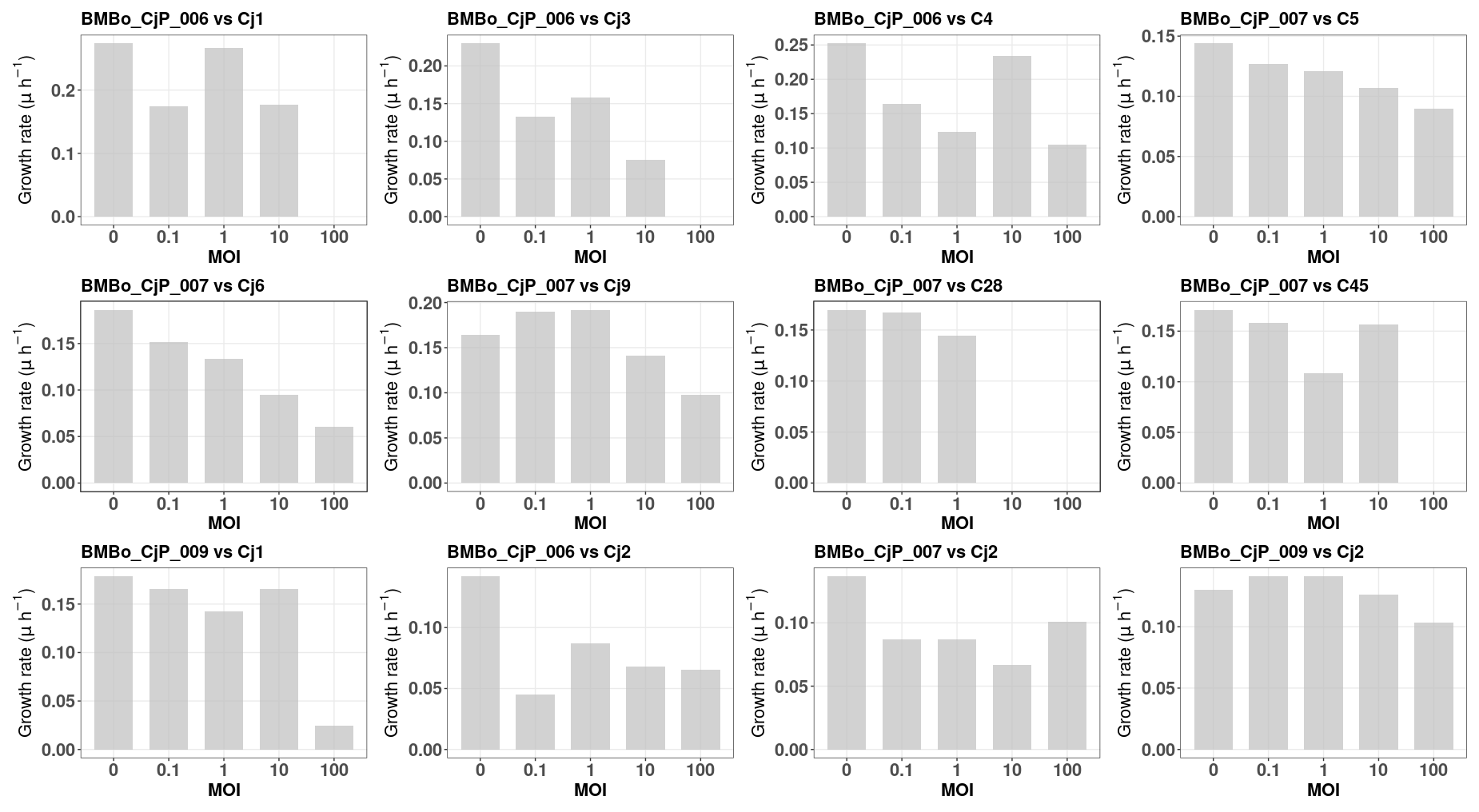


**Supplementary Figure 3.** Growth rate μ (h^-1^) vs. MOI for each growth kinetic, determined at different MOI (100, 10, 1, and 0.1). MOI 0 represents the positive control (bacterial growth without bacteriophage added).

## Supplementary Tables

**Supplementary Table 1.** Bacteriophage host range, phenotypic resistance profiles, and source of *C. jejuni* and *Campylobacter* isolates. Coordinates: Informal market (16 de Julio): -16.497338, -68.176240. Informal market (Mercado Uruguay): -16.497442, -68.143624. Public square (Plaza Murillo) -16.495760, -68.133549

| **Bacterial Isolate** | **Bacteriophage host range** | | | **Phenotypic resistance profile** | **Acquired AMR genes** | **Chromosomal**  **mutations mediating AMR** | **Source** | **Sampling site** |
| --- | --- | --- | --- | --- | --- | --- | --- | --- |
|  | **BMBo_CjP_006** | **BMBo_CjP_007** | **BMBo_CjP_009** |  |  |  |  |  |
| Cj1 | + | - | + | AMP, CHL, NAL | *bla*OXA-61 | *gyrA* (T86I)  *cmeABR* | Chicken feces | Informal market (16 de julio) |
| Cj2 | + | + | + | AMP, AMC, CHL, NAL, SXT | *bla*OXA-61 | *gyrA* (T86I)  *cmeABR* | Chicken feces | Informal market (16 de julio) |
| Cj3 | + | - | - | AMP, TET, CHL, CIP, NAL | *bla*OXA-61 | *gyrA* (T86I)  *cmeABR* | Chicken feces | Informal market (16 de julio) |
| C4 | + | - | - | AMP, CEP, TET, CHL, NAL |  |  | Chicken feces | Informal market (16 de julio) |
| C5 | - | + | - | CHL, NAL |  |  | Chicken feces | Informal market (16 de julio) |
| Cj6 | - | + | - | AMP, CHL, CIP | *bla*OXA-61 *tet*(O) | *gyrA* (T86I)  *cmeABR* | Chicken feces | Informal market (16 de julio) |
| C7 | - | - | - | AMP, CEP, CIP, NAL |  |  | Chicken feces | Informal market (16 de julio) |
| C8 | - | - | - | ERY |  |  | Chicken feces | Informal market (16 de julio) |
| Cj9 | - | + | - | AMP, CIP | *bla*OXA-61 | *gyrA* (T86I)  *cmeABR* | Chicken feces | Informal market (16 de julio) |
| Cj10 | - | - | - | AMP, ERY, CHL, CIP | *bla*OXA-61 | *gyrA* (T86I)  *cmeABR* | Chicken feces | Informal market (16 de julio) |
| C11 | - | - | - | AMP, CHL, NAL |  |  | Chicken feces | Informal market (16 de julio) |
| C12 | - | - | - | TET, ERY, CHL, CIP, NAL, SXT |  |  | Chicken feces | Backyard farm (El Alto city) |
| C13 | - | - | - | AMP, ERY, CHL, CIP, NAL, SXT |  |  | Chicken feces | Backyard farm (El Alto city) |
| C14 | - | - | - | CHL, CIP, NAL, SXT |  |  | Chicken feces | Backyard farm (El Alto city) |
| C15 | - | - | - | ERY, CIP, NAL, SXT |  |  | Chicken feces | Backyard farm (El Alto city) |
| C16 | - | - | - | AMP, ERY, CHL, CIP, NAL, SXT |  |  | Chicken feces | Backyard farm (El Alto city) |
| Cj17 | - | - | - | ERY, CHL, CIP, NAL, SXT | *bla*OXA-61  *tet*(O) | *gyrA* (T86I)  *cmeABR* | Chicken feces | Backyard farm (El Alto city) |
| Cj18 | - | - | - | AMP, CHL, NAL | *bla*OXA-61  *tet*(O) | *gyrA* (T86I)  *rpsL*  *porA*  *cmeABR* | Chicken feces | Backyard farm (El Alto city) |
| C19 | - | - | - | AMP, AMC, CHL, NAL, SXT |  |  | Free-living birds (Pigeon) feces | Public square (Plaza Murillo) |
| Cj20 | - | - | - | AMP, TET, CHL, CIP, NAL | *bla*OXA-61  *tet*(O) | *gyrA* (T86I)  *cmeABR* | Chicken feces | Informal market (Mercado Uruguay) |
| C21 | - | - | - | AMP, CEP, TET, CHL, NAL |  |  | Chicken feces | Informal market (Mercado Uruguay) |
| C22 | - | - | - | CHL, NAL |  |  | Sludge from  farm | Backyard farm (El Alto city) |
| Cj23 | - | - | - | AMP, CHL, CIP | *bla*OXA-61  *tet*(O) | *gyrA* (T86I)  *cmeABR* | Sludge from  farm | Backyard farm (El Alto city) |
| Cj24 | - | - | - | AMP, CEP, CIP, NAL | *bla*OXA-61  *tet*(O) | *gyrA* (T86I)  *cmeABR* | Chicken feces | Backyard farm (El Alto city) |
| C25 | - | - | - | ERY |  |  | Chicken feces | Backyard farm (El Alto city) |
| C26 | - | - | - | AMP, CIP |  |  | Chicken feces | Informal market (16 de julio) |
| C27 | - | - | - | AMP, CHL, CIP |  |  | Chicken feces | Informal market (16 de julio) |
| C28 | - | + | - | AMP, ERY, CHL, CIP, NAL |  |  | Chicken feces | Informal market (16 de julio) |
| C29 | - | - | - | AMP, TET, CHL, CIP, NAL |  |  | Chicken feces | Informal market (16 de julio) |
| C30 | - | - | - | AMP, CEP, TET, CIP, NAL |  |  | Chicken feces | Informal market (16 de julio) |
| C31 | - | - | - | CHL, NAL |  |  | Free-living birds (pigeon) feces | Public square (Plaza Murillo) |
| C32 | - | - | - | AMP, CHL, CIP |  |  | Chicken feces | Informal market (16 de julio) |
| C33 | - | - | - | AMP, CEP, CIP, NAL |  |  | Chicken feces | Informal market (16 de julio) |
| Cj34 | - | - | - | AMP, AMC, CEP, TET, CHL, CIP, NAL, SXT | *bla*OXA-61  *tet*(O) | *gyrA* (T86I)  *cmeABR* | Chicken feces | Informal market (16 de julio) |
| C35 | - | - | - | AMP, AMC, CEP, TET, CHL, CIP, NAL, SXT |  |  | Sludge from  farm | Backyard farm (El Alto city) |
| C36 | - | - | - | AMP, CHL, NAL, SXT |  |  | Chicken feces | Backyard farm (El Alto city) |
| C37 | - | - | - | AMP, CEP, CHL, NAL, SXT |  |  | Chicken feces | Backyard farm (El Alto city) |
| C38 | - | - | - | AMP, AMC, TET, CHL, CIP, NAL, SXT |  |  | Free-living birds (pigeon) feces | Public square (Plaza Murillo) |
| C39 | - | - | - | AMP, CIP, NAL, SXT |  |  | Free-living feces | Public square (Plaza Murillo) |
| C40 | - | - | - | AMP, CEP, TET, ERY, CIP, NAL, SXT |  |  | Free-living feces | Public square (Plaza Murillo) |
| C41 | - | - | - | CEP, ERY, CIP, NAL, SXT |  |  | Chicken feces | Informal market (16 de julio) |
| C42 | - | - | - | AMP, NAL, SXT |  |  | Chicken feces | Informal market (16 de julio) |
| Cj43 | - | - | - | AMP, CEP, CHL, NAL, SXT | *bla*OXA-61 *tet*(O) | *gyrA* (T86I)  *cmeABR* | Chicken feces | Informal market (16 de julio) |
| C44 | - | - | - | CHL, CIP, NAL, SXT |  |  | Chicken feces | Informal market (16 de julio) |
| C45 | - | + | - | AMP, AMC, CEP, CIP, NAL, SXT |  |  | Chicken feces | Informal market (16 de julio) |
| C46 | - | - | - | AMP, TET, CIP, NAL, SXT |  |  | Chicken feces | Informal market (16 de julio) |
| C47 | - | - | - | AMP, TET, SXT |  |  | Chicken feces | Informal market (16 de julio) |
| Cj48 | - | - | - | AMP, SXT | *bla*OXA-452  *tet*(O) | *gyrA* (T86I)  *cmeABR* | Chicken feces | Informal market (16 de julio) |
| C49 | - | - | - | AMP, SXT |  |  | Chicken feces | Informal market (16 de julio) |
| C50 | - | - | - | AMP, CIP, NAL, SXT |  |  | Chicken feces | Informal market (16 de julio) |
| C51 | - | - | - | AMP, TET, CIP, NAL, SXT |  |  | Chicken feces | Informal market (16 de julio) |
| C52 | - | - | - | AMP, TET, CIP, NAL, SXT |  |  | Chicken feces | Informal market (16 de julio) |
| C53 | - | - | - | AMP, TET, CIP, NAL, SXT |  |  | Chicken feces | Informal market (16 de julio) |
| C54 | - | - | - | AMP, AMC, TET, CIP, NAL, SXT |  |  | Chicken feces | Informal market (16 de julio) |
| C55 | - | - | - | AMP, SXT |  |  | Chicken feces | Informal market (16 de julio) |

**Supplementary Table 2.** Functional annotation of sequenced bacteriophage genomes

| **Gene description** | **BMBo_CjP_006** | **BMBo_CjP_007** | **BMBo_CjP_009** |
| --- | --- | --- | --- |
| **CDS** | 146 | 144 | 90 |
| **Connector** | 0 | 0 | 1 |
| **DNA, RNA and nucleotide metabolism** | 17 | 17 | 9 |
| **Bacteriophage head and packaging** | 6 | 5 | 8 |
| **Integration and excision** | 0 | 0 | 0 |
| **Lysis** | 3 | 3 | 2 |
| **Moron, auxiliary metabolic gene and host takeover** | 4 | 4 | 1 |
| **Other** | 8 | 8 | 2 |
| **Tail** | 5 | 5 | 12 |
| **Transcription regulation** | 1 | 1 | 0 |
| **Unknown function** | 102 | 101 | 55 |
| **tRNAs** | 0 | 0 | 0 |
| **CRISPRs** | 0 | 0 | 0 |
| **tmRNAs** | 0 | 0 | 0 |
| **VFDB Virulence factors** | 0 | 0 | 0 |
| **CARD antimicrobial resistance genes** | 0 | 0 | 0 |
